# Susceptibility of *Salmonella* serotypes isolated from meat and meat contact surfaces to thermal, acidic, and alkaline treatments and disinfectants

**DOI:** 10.1002/fsn3.3221

**Published:** 2023-01-09

**Authors:** Leila Manafi, Javad Aliakbarlu, Habib Dastmalchi Saei

**Affiliations:** ^1^ Faculty of Veterinary Medicine, Department of Food Hygiene and Quality Control Urmia University Urmia Iran; ^2^ Faculty of Veterinary Medicine, Department of Microbiology Urmia University Urmia Iran

**Keywords:** acidic, antibiotic, biofilm, disinfectant, *Salmonella*, thermal

## Abstract

The present study was conducted to evaluate the response of 29 *Salmonella* isolates to exposure to thermal (60°C for 2 min), acidic (pH 2.9 for 30 min), and alkaline (pH 11 for 60 min) treatments and investigate the susceptibility of the isolates and their biofilms to disinfectants. The reductions of *Salmonella* isolates populations subjected to each treatment were analyzed according to their isolation source, serotype, antibiotic resistance pattern, and biofilm formation ability. Median reductions for all of *Salmonella* isolates populations after thermal, acidic, and alkaline treatments were 1.8, 2.1, and 0.7 log CFU/ml, respectively. The isolates behavior under stress conditions were not related to their isolation source, serotype, or biofilm formation ability. The median reduction after alkaline treatment in non‐MDR (multidrug‐ resistant) isolates populations was significantly (*p* < .05) higher than MDR isolates. The median reduction in biofilms of moderate biofilm producers by disinfectants was significantly (*p* < .05) higher than that of strong biofilm producers. In conclusion, *Salmonella* isolates showed the highest susceptibility to acidic treatment and MDR isolates were more resistant to alkaline treatment than non‐MDR ones. The current study also revealed that the strong biofilm producer isolates were more resistant to disinfectants than moderate biofilm producers. This study facilitated the understanding of the relationship between *Salmonella* characteristics (isolation source, serotype, antibiotic resistance pattern, and biofilm formation ability) and its susceptibility to thermal, acidic, and alkaline treatments and disinfectants. The findings are helpful for the prevention and control of *Salmonella*.

## Graphical abstract

Twenty‐nine *Salmonella* isolates were subjected to various treatments. *Salmonella* isolates showed the highest susceptibility to acidic treatment. MDR isolates were more resistant to alkaline treatment than non‐MDR ones. The strong biofilm producers were more resistant to disinfectants than moderate biofilm producers.

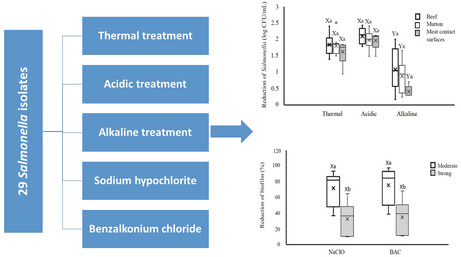

## 1 INTRODUCTION

Food‐borne diseases are a serious public health problem due to their significant effect on the health and economy of society (Tafida et al., 2013; Park et al., 2020). *Salmonella* strains are one of the major reasons for food‐borne disease outbreak worldwide (Huang et al., 2019). The outbreak usually occurs due to the consumption of meat (undercooked) and other contaminated food of animal origin (Gill et al., 2019; Hu et al., 2020; Jaja et al., 2019; Yang et al., 2020). Despite the many health measures taken during the meat production, food‐borne illnesses still occur, and complete prevention of bacterial contamination of carcass is difficult due to the transfer of bacteria from the skin and intestine of the animal to the muscle tissue during slaughter and carcass cutting. Various interventions have been performed to reduce the bacterial contamination of beef, including washing carcass skin with an alkaline solution, pasteurization with hot water, and acidic treatment of carcass with organic acids (Gill et al., 2019; Loretz et al., 2011).

Biofilms can represent an important source of contamination in food processing facilities, leading to contamination in foods. Then, improper disinfection and cleaning process may result in outbreaks of food‐borne diseases (Loretz et al., 2011). It has been shown that bacteria within biofilms are more resistant to environmental stresses and processing than planktonic cells (Hu et al., 2020; Kocot & Olszewska, 2020; Zhang, 2020), and probably represent an important reservoir for cross‐contamination in the food industry (Purohit et al., 2020). According to the previous studies, *Salmonella* species are capable of forming biofilms under various conditions on food processing surfaces that increase their prevalence and survival (Dantas et al., 2020; Hu et al., 2020; Lim et al., 2017). Several studies have reported that *Salmonella* contamination was resistant to disinfectants, and often remained in the environment after disinfection (Hu et al., 2020; Soni et al., 2013; Wang, Zhang et al., 2015).

Some previous studies evaluated *Salmonella* susceptibility to decontamination interventions (Gill et al., 2019; Loretz et al., 2011). However, much less studies have evaluated the relationship between *Salmonella* characteristics (isolation source, serotype, antibiotic resistance pattern, and biofilm formation ability) and its susceptibility to thermal, acidic, and alkaline treatments and disinfectants. Therefore, this study was performed with the following aims: (1) Evaluation of the response of *Salmonella* serotypes isolated from different sources (beef, mutton, and meat contact surfaces) to thermal, acidic, and alkaline treatments; (2) Investigation of the sensitivity of the planktonic cells of *Salmonella* isolates and their biofilms to selective disinfectants; (3) Determination whether the reductions after decontamination interventions are related to isolation sources, serotypes, antibiotic resistant patterns, or biofilm formation ability.

## 2 MATERIALS AND METHODS

### 2.1 Bacterial strains

A total of 29 *Salmonella* isolates were used in this study. The characteristics of *Salmonella* isolates are given in Table 1 (Manafi et al., 2020).

**TABLE 1:** The characteristics of the *Salmonella* serotypes isolated from meat and meat contact surfaces in Urmia, Iran

Serotypes identified	Source of isolation	Antibiotic resistance	Biofilm formation category
*Salmonella* Enteritidis	Beef	Not‐MDR	Moderate
*Salmonella* Enteritidis	Beef	Not‐MDR	Strong
*Salmonella* Enteritidis	Beef	Not‐MDR	Moderate
*Salmonella* Enteritidis	Beef	MDR	Moderate
*Salmonella* Enteritidis	Beef	Not‐MDR	Moderate
*Salmonella* Enteritidis	Mutton	Not‐MDR	Moderate
*Salmonella* Enteritidis	Meat contact surface	MDR	Strong
*Salmonella* Typhimurium	Beef	Not‐MDR	Moderate
*Salmonella* Typhimurium	Mutton	Not‐MDR	Moderate
*Salmonella* Typhimurium	Mutton	Not‐MDR	Moderate
*Salmonella* Typhi	Beef	MDR	Moderate
*Salmonella* Typhi	Mutton	Not‐MDR	Moderate
*Salmonella* spp.	Beef	MDR	Strong
*Salmonella* spp.	Beef	MDR	Moderate
*Salmonella* spp.	Beef	MDR	Moderate
*Salmonella* spp.	Beef	Not‐MDR	Moderate
*Salmonella* spp.	Beef	MDR	Moderate
*Salmonella* spp.	Beef	Not‐MDR	Moderate
*Salmonella* spp.	Beef	Not‐MDR	Strong
*Salmonella* spp.	Mutton	MDR	Moderate
*Salmonella* spp.	Mutton	Not‐MDR	Moderate
*Salmonella* spp.	Mutton	Not‐MDR	Strong
*Salmonella* spp.	Mutton	MDR	Strong
*Salmonella* spp.	Mutton	Not‐MDR	Moderate
*Salmonella* spp.	Mutton	MDR	Moderate
*Salmonella* spp.	Meat contact surface	MDR	Moderate
*Salmonella* spp.	Meat contact surface	MDR	Moderate
*Salmonella* spp.	Meat contact surface	MDR	Moderate
*Salmonella* spp.	Meat contact surface	MDR	Strong

### 2.2 Preparation of the bacterial suspension

The bacterial cells were initially sub‐cultured from frozen stocks in trypticasein soy (TS) broth (BioMaxima S.A, Poland) without glucose and incubated at 37°C for 18–24 h. After incubation, a loop from the TS broth was streaked onto TS agar (Liofilchem, Teramo, Italy) and incubated at 37°C for 24 h. Next, single colonies were chosen and inoculated in 10 ml of TS broth and then incubated at 37°C for 24 h. After that, the bacterial suspensions were centrifuged (Hettich, Tuttlingen, Germany) at 4226*g* for 10 min. The supernatant was removed, and the bacterial pellet was suspended in 10 ml of phosphate‐buffered saline (PBS; pH 7.00). The suspension was re‐centrifuged at the above‐mentioned conditions. Finally, 10 ml of the 4°C TS broth was added to the bacterial pellet, and the optical density (OD) of the cell suspensions was adjusted to 0.1 (approximately 10^8^ CFU/ml) at 600 nm using a spectrophotometer (Pharmacia LKB, Uppsala, Sweden). The cell suspensions were then stored at 4°C for 48 h before use for testing (cold suspension). On the day of the experiment, serial dilutions of up to 10^−5^ in PBS were prepared from the cold cell suspensions, and 100 μl of the last dilution was cultured on TS agar and incubated at 35°C for 48 h. Finally, the colonies grown on the agar were counted and taken as time‐zero values (Gill et al., 2019).

### 2.3 Evaluation of the response of *Salmonella* isolates to thermal, acidic, and alkaline treatments

This experiment was designed according to the description of Gill et al. (2019) with a slight modification. The treatment conditions (especially exposure time) were examined through the preliminary experiments on five *Salmonella* isolates and the conditions which resulted in countable colony were selected. The selected treatment conditions were 60°C for 2 min, 5% lactic acid (pH 2.9) for 30 min, and pH 11 for 60 min.

### 2.4 Thermal treatment

*Salmonella* isolates were exposed to 60°C for 2 min to evaluate their response to thermal treatment. At first, 100 μl of the vortexed cold suspension was added to 9.9 ml of 60°C TS broth and placed in a water bath at the same temperature for 2 min. After treatment, the tube containing suspension was vortexed, and 500 μl of the treated suspension added to the test tube containing 4.5 ml of PBS pre‐cooled in the ice‐water bath. The tube was diluted to 10^−3^ in PBS, and then 100 μl of the treated suspension and each of dilutions was cultured onto TS agar and incubated at 35°C for 48 h (Gill et al., 2019). All steps (inoculation, dilution, plating, and colony counting) were also conducted on untreated controls.

### 2.5 Acidic and alkaline treatments

To prepare the acidic and alkaline treatments, lactic acid or NaOH was added to TS broth, respectively. The acidic TS broth solution was prepared by adding distilled water and 90% lactic acid to double strength TS broth to produce a 5% lactic acid TS solution with a pH of 2.9. The alkaline TS broth solution was also prepared by adding 5 N NaOH to double strength TS broth to create a pH 11.0 TS solution. The solutions were sterilized by filtration (0.22 μm) and stored at 4°C until use.

A 100 μl aliquot of the cold OD adjusted suspension was added to the acidic and alkaline TS broth solutions. After vortexing, the solutions were stored at 4°C for 30 min (acidic) and 60 min (alkaline) to apply the treatments. Subsequently, the tubes containing the treated suspension were vortexed and diluted up to 10^−3^ in PBS, and then 100 μl of each dilution was cultured onto TS agar, followed by incubation at 35°C for 48 h (Gill et al., 2019). TS broth (pH 7.3) inoculated with the cold suspension was used as untreated control. All steps were also performed on untreated controls.

### 2.6 Colony counts

After each treatment and at the end of the incubation period, the colony‐forming units (CFU) on the plates were enumerated, and the results converted to log CFU/ml. Then, the mean log CFU/ml was calculated for untreated control and treatments. The log reduction for each treatment was computed according to the following equation:

$$\mathrm{Log}\;\text{reduction}=\log\;\mathrm{CFU}/{\mathrm{ml}}_{\text{control}}–\log\;\mathrm{CFU}/{\mathrm{ml}}_{\text{treatment}}$$

### 2.7 Susceptibility of *Salmonella* isolates to disinfectants

The susceptibility of *Salmonella* isolates to sodium hypochlorite (NaClO, Active chlorine 6–14%, Merck, Germany) and benzalkonium chloride (BAC 20%, Fazel Derakhshan Co., Tehran, Iran) was tested by agar well diffusion method. Initially, 3–5 colonies of the isolates were inoculated into TS broth and incubated at 37°C for 18–24 h. Then, the OD of bacterial suspension was adjusted to 0.1 at 600 nm using a spectrophotometer and cultured onto Mueller–Hinton agar (Mirmedia, Tehran, Iran) plate using a sterile swab. An 8 mm well was created in the center of the plate with a sterile cork‐borer and filled with 100 μl of each disinfectant (500 mg/L BAC and 2000 mg/L NaClO), and then incubated at 37°C for 24 h. Finally, the inhibition zones were recorded in millimeters on each plate (Lim et al., 2017).

### 2.8 Susceptibility of *Salmonella* biofilms to disinfectants

Assessment of the effect of disinfectants on *Salmonella* biofilm was performed in a 96‐well microplate according to the previously described method with some modifications (Lim et al., 2017). At first, 180 μl of TS broth was dispensed in wells of the microplate, then 20 μl of overnight culture (OD = 0.1 at 600 nm) was added to each well (ten replicate for each disinfectant), and incubated at 37°C for 48 h. The microplate content was emptied by inverting and then washed three times with sterile PBS. After washing, 200 μl of the disinfectant (50 mg/L NaClO and 100 mg/L BAC) were added to six wells, and the microplate was incubated at room temperature for 10 min. Also, 200 μl of the PBS was added to the four wells as the control wells. Following incubation, the microplate content was discharged, and 200 μl neutralizing broth (Prepared according to the method described by Vázquez‐Sánchez et al., 2014) was added. After 5 min, the wells were washed with PBS and then dried and stained with 200 μl crystal violet (1%) for 30 min. The content of the wells was evacuated and washed with running water, and then dried at room temperature. Finally, 200 μl ethanol (96%) was distributed in the wells and after 20 min, the OD was measured using a microplate reader system (DANA, DA3200, Iran) at 570 nm. The reduction rate of biofilm (%) was computed according to the formula below:

$$\text{Biofilm reduction}\;\left(\%\right)=\frac{\left(C-B\right)-\left(T-B\right)}{\left(C-B\right)}\times 100$$

where C = mean OD_570 nm_ of the control wells (TS broth+ bacteria+ PBS), B = mean OD_570 nm_ of the wells containing TS broth, and T = mean of OD_570 nm_ of treatment wells (TS broth+ bacteria+ disinfectant).

### 2.9 Statistical analysis

The experiments were performed in triplicate and repeated twice for each isolate on different days. The bacterial reductions were statistically analyzed according to the following criteria: (a) type of treatment (thermal, acidic, alkaline, NaClO, and BAC); (b) source of the isolation (beef, mutton, and the meat contact surfaces); (c) serotype (*Salmonella* Enteritidis, *Salmonella* Typhimurium, *Salmonella* Typhi, and the other *Salmonella* serotypes); (d) biofilm formation ability (moderate and strong biofilm producers) and antibiotic resistance pattern (not‐MDR and MDR). According to the results of the Shapiro test (IBM SPSS Statistics 26, IBM Corp., Armonk, NY, USA), some data sets in this study were not normally distributed. Therefore, the median values were compared using Kruskal–Wallis (k samples) and Mann–Whitney U (2 samples) nonparametric tests, at significance level of 0.05. Also, a Pearson correlation test (IBM SPSS Statistics 26, USA) was used to determine the correlation between different treatments.

## 3 RESULTS

### 3.1 Response of *Salmonella* isolates to thermal, acidic, and alkaline treatments

The response of the *Salmonella* isolates populations to the treatments was measured by log reduction and presented in Table 2. Variability, expressed by the interquartile range (IQR) and coefficient of variation (CV), was due to differences in the treatments. The range of log reductions was from 0.9 to 2.5 log CFU/ml for the thermal treatment, from 1.4 to 2.4 log CFU/ml for acidic treatment, and 0.1 to 1.9 log CFU/ml for alkaline treatment. Meanwhile, the highest median log reduction (2.1) was obtained after acidic treatment, followed by thermal treatment (1.8), while alkaline treatment had the lowest median log reduction (0.7). There were significant differences (*p* < .05) among median log reductions induced by all three treatments.

**TABLE 2:** *Salmonella* reductions (log CFU/ml) after exposure to thermal (60°C for 2 min), acid (pH 2.9 for 30 min), and alkaline (pH 11 for 60 min) treatments

	*n*	Range (IQR)			Median			CV		
		Thermal	Acidic	Alkaline	Thermal	Acidic	Alkaline	Thermal	Acidic	Alkaline
*Salmonella*	29	0.9–2.5 (0.32)	1.4–2.4 (0.36)	0.1–1.9 (0.91)	1.8^a^	2.1^b^	0.7^c^	17	12	63

*Note*: a–c: Different superscript small letters indicate statistically significant differences (*p* < .05) among median log reductions induced by treatments.

Abbreviations: CV, coefficient of variation; IQR, interquartile range.

**FIGURE 1:**
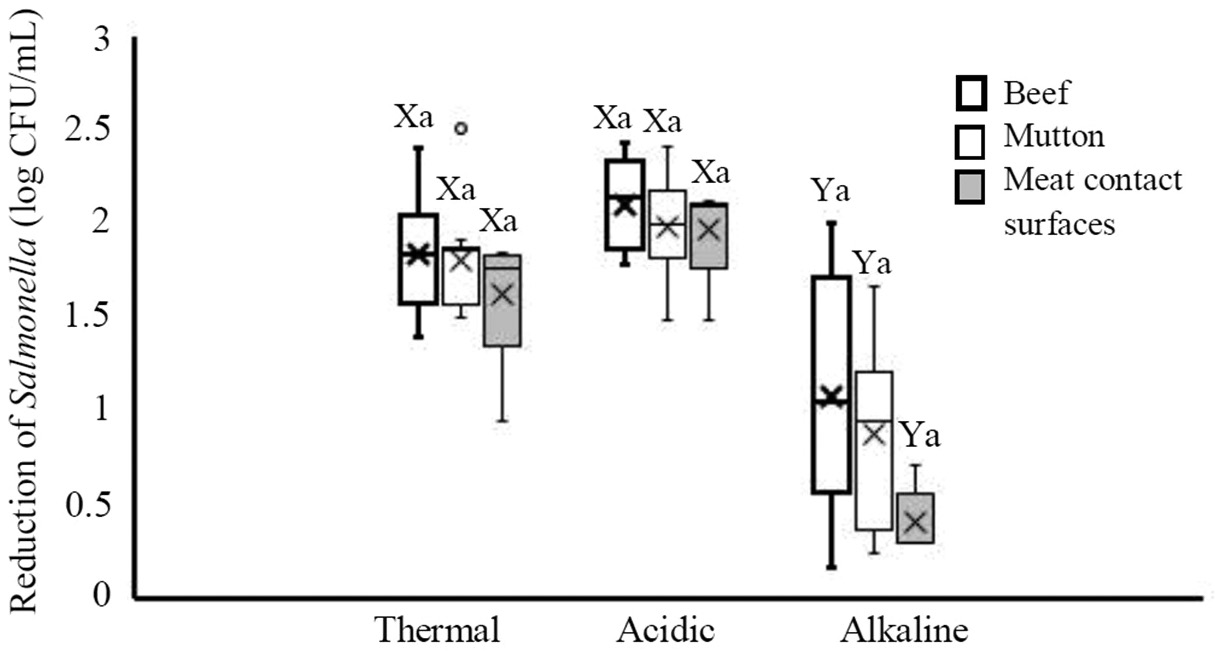
Reduction of *Salmonella* isolated from beef, mutton, and meat contact surfaces after exposure to thermal (60°C for 2 min), acid (pH 2.9 for 30 min), and alkaline (pH 11 for 60 min) treatments. Different small letters indicate significant differences (*p* < .05) in median reductions within a treatment. Different capital letters indicate significant differences (*p* < .05) in median reductions across treatments. Boxes show data within the first and third quartile (interquartile range, IQR), central horizontal lines show medians, x marks show means, and whiskers show the lowest data point within 1.5 IQR of the first quartile, and the highest data point within 1.5 IQR of the third quartile. Open circles show data points above or below the 1.5 IQR of the first or third quartile.

Besides, the bacterial reductions under treatments were statistically analyzed according to the source of the isolation, serotype, biofilm formation ability, and antibiotic resistance pattern of the isolates. In this regard, the log reduction of *Salmonella* isolates populations from different sources following exposure to three treatments is shown in Figure 1. The highest median log reduction (2.14 log CFU/ml) was induced by acidic treatment in *Salmonella* populations isolated from beef, while the lowest reduction (0.31 log CFU/ml) was caused by alkaline treatment in *Salmonella* isolated from meat contact surfaces. According to statistical analyses, the median log reductions of *Salmonella* from all three sources after acidic treatment were similar to those after thermal treatment (*p* > .05) but were significantly higher than those after alkaline treatment (*p* < .05). However, there was no significant difference among the median log reduction of the three *Salmonella* sources in any of the treatment groups (*p* > .05).

**FIGURE 2:**
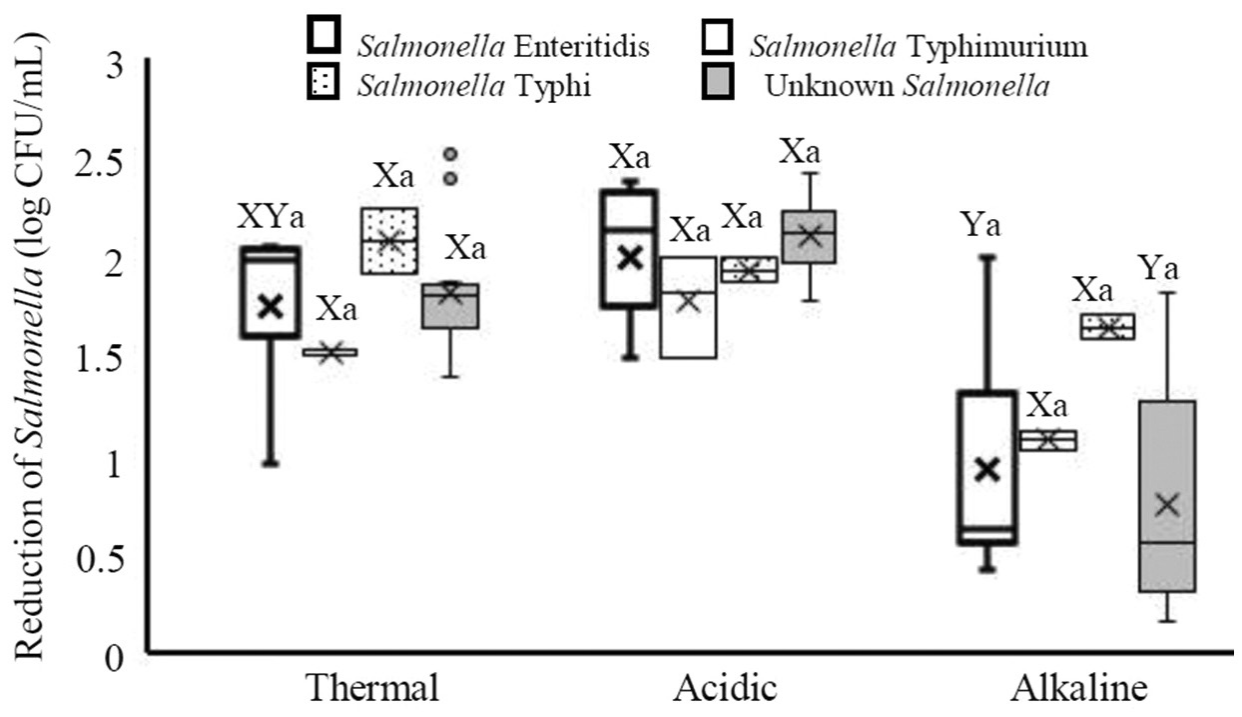
Reduction of *Salmonella* serotypes including *Salmonella* Enteritidis, *Salmonella* Typhimurium, *Salmonella* Typhi, and unknown *Salmonella* after exposure to thermal (60°C for 2 min), acidic (pH 2.9 for 30 min), and alkaline (pH 11 for 60 min) treatments. Different small letters indicate significant differences (*p* < .05) in median reductions within a treatment. Different capital letters indicate significant differences (*p* < .05) in median reductions across treatments. Boxes show data within the first and third quartile (interquartile range, IQR), central horizontal lines show medians, x marks show means, and whiskers show the lowest data point within 1.5 IQR of the first quartile, and the highest data point within 1.5 IQR of the third quartile. Open circles show data points above or below the 1.5 IQR of the first or third quartile.

**FIGURE 3:**
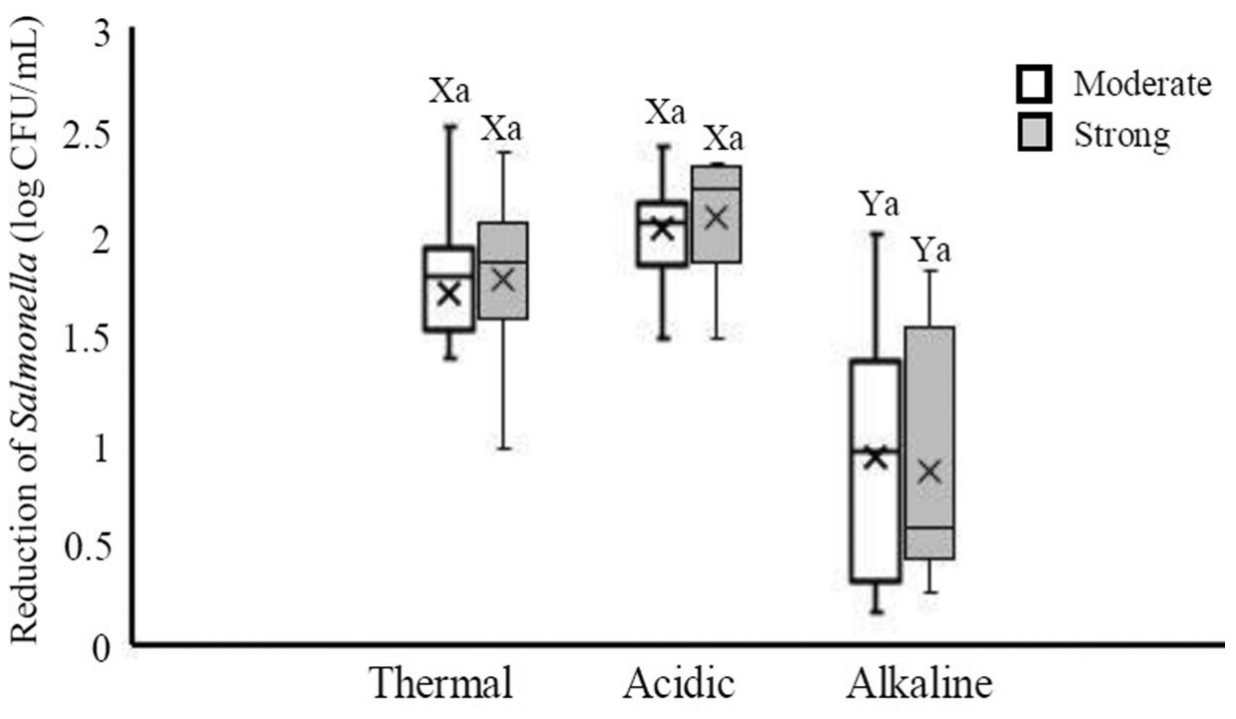
Reduction of *Salmonella* isolates with the ability of moderate biofilm produce, and those strong biofilm producer after exposure to thermal (60°C for 2 min), acidic (pH 2.9 for 30 min), and alkaline (pH 11 for 60 min) treatments. Different small letters indicate significant differences (*p* < .05) in median reductions within a treatment. Different capital letters indicate significant differences (*p* < .05) in median reductions across treatments. Boxes show data within the first and third quartile (interquartile range, IQR), central horizontal lines show medians, x marks show means, and whiskers show the lowest data point within 1.5 IQR of the first quartile, and the highest data point within 1.5 IQR of the third quartile.

Furthermore, the response of different serotypes of *Salmonella* to the treatments was analyzed and the results are presented in Figure 2. The highest median log reduction (2.14 log CFU/ml) was induced in *Salmonella* Enteritidis by acidic treatment, while the lowest reduction (0.55 log CFU/ml) was found in unknown *Salmonella* group exposed to alkaline treatment. The median reductions of *S*. Enteritidis after acidic treatment were significantly higher than those after alkaline treatment (*p* < .05). Besides, the median reductions of other unknown *Salmonella* after acidic and thermal treatments were significantly higher than those after alkaline treatment (*p* < .05). However, the median reductions among serotypes were not significant (*p* > .05) in none of the treatments. The *Salmonella* isolates were grouped based on their biofilm formation abilities and their responses to thermal, acidic, and alkaline treatments were evaluated (Figure 3). It was found that the best treatment against both moderate and strong biofilm producer *Salmonella* was acidic treatment. The median log reductions induced by acidic treatment in moderate and strong biofilm producer isolates were 2.05 and 2.22 log CFU/ml, respectively. It was also found that alkaline treatment is not an appropriate treatment for decreasing *Salmonella*. In both moderate and strong biofilm producer isolates, the log median reductions following acidic and thermal treatments were significantly higher than those after alkaline treatment (*p* < .05). However, there was no difference between median reduction after acid treatment and those after thermal treatment (*p* > .05).

**FIGURE 4:**
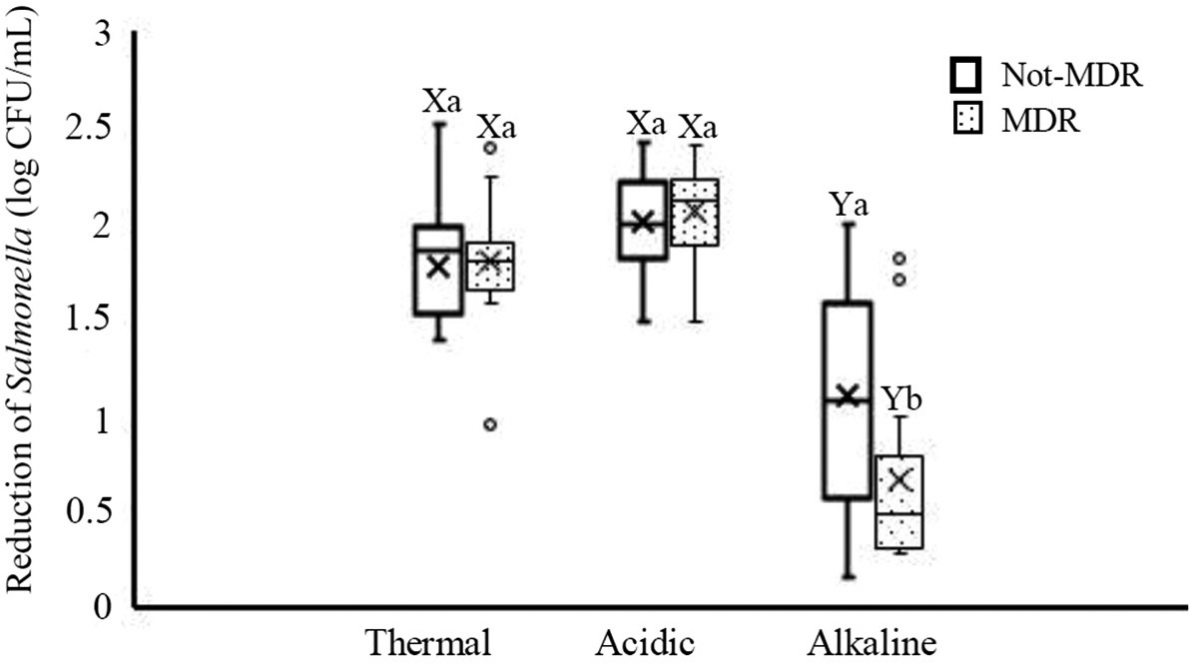
Reduction of MDR (multi drug resistant) and not‐MDR *Salmonella* isolates after exposure to thermal (60°C for 2 min), acidic (pH 2.9 for 30 min) and alkaline (pH 11 for 60 min) treatments. Different small letters indicate significant differences (*p* < .05) in median reductions within a treatment. Different capital letters indicate significant differences (*p* < .05) in median reductions across treatments. Boxes show data within the first and third quartile (interquartile range, IQR), central horizontal lines show medians, x marks show means, and whiskers show the lowest data point within 1.5 IQR of the first quartile, and the highest data point within 1.5 IQR of the third quartile. Open circles show data points above or below the 1.5 IQR of the first or third quartile.

Figure 4 shows the response of MDR and not‐MDR *Salmonella* isolates to thermal, acidic, and alkaline treatments. It was found that the acidic treatment with approximately 2 log reduction was the best treatment against both MDR and not‐MDR isolates. Meanwhile, MDR isolates showed the highest resistance to alkaline treatment. The difference in the median reductions between the not‐MDR and MDR isolates after the alkaline treatment was significant (*p* < .05). However, no significant difference (*p* > .05) in the median reductions between the not‐MDR and MDR isolates was noted after acidic and thermal treatments. Overall, the median reductions in both not‐MDR and MDR groups after the acidic and thermal treatments were significantly higher than those after alkaline treatment (*p* < .05).

### 3.2 Susceptibility of the isolates to disinfectants

**FIGURE 5:**
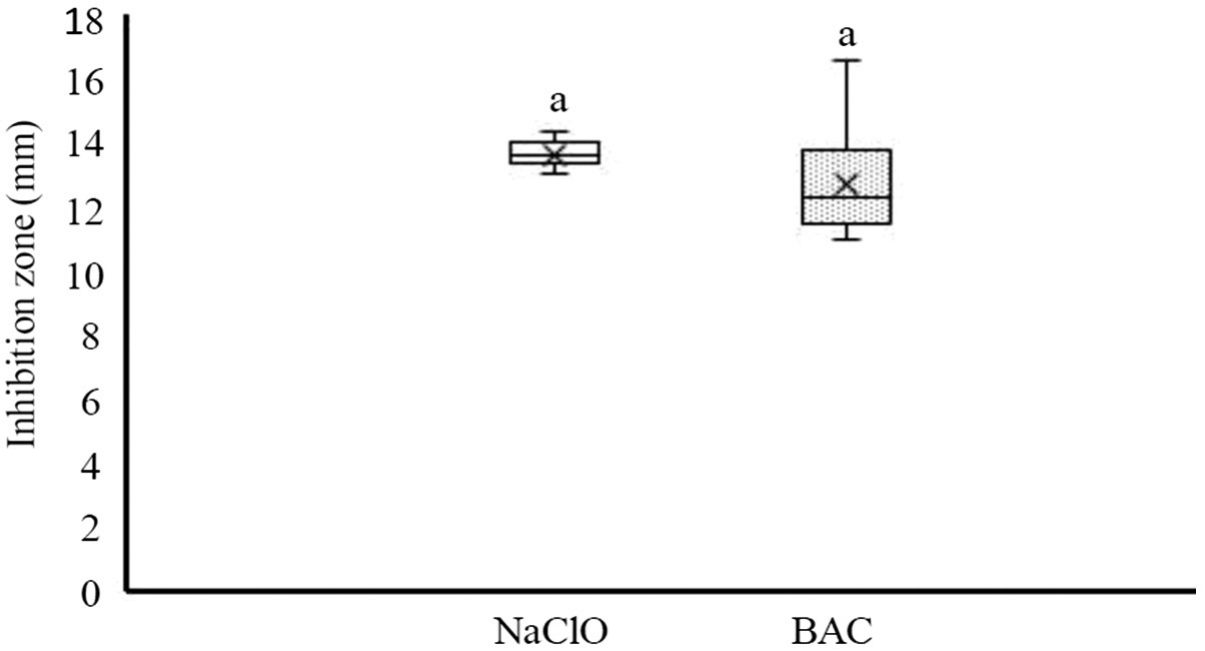
The susceptibility of *Salmonella* isolates to sodium hypochlorite (NaClO) and benzalkonium chloride (BAC). Inhibition zones that share a common letter are not significantly different (*p* > .05). Boxes show data within the first and third quartile (interquartile range, IQR), central horizontal lines show medians, x marks show means, and whiskers show the lowest data point within 1.5 IQR of the first quartile, and the highest data point within 1.5 IQR of the third quartile.

The agar well diffusion method was carried out to evaluate the susceptibility of the suspension of the isolates to NaClO and BAC. The inhibition zones for NaClO and BAC ranged from 13.07 to 14.35 mm and 11.02 to 16.63 mm, respectively (Figure 5). There was no significant difference in the susceptibility of *Salmonella* isolates to NaClO and BAC (*p* > .05).

### 3.3 Susceptibility of *Salmonella* biofilms to disinfectants

**FIGURE 6:**
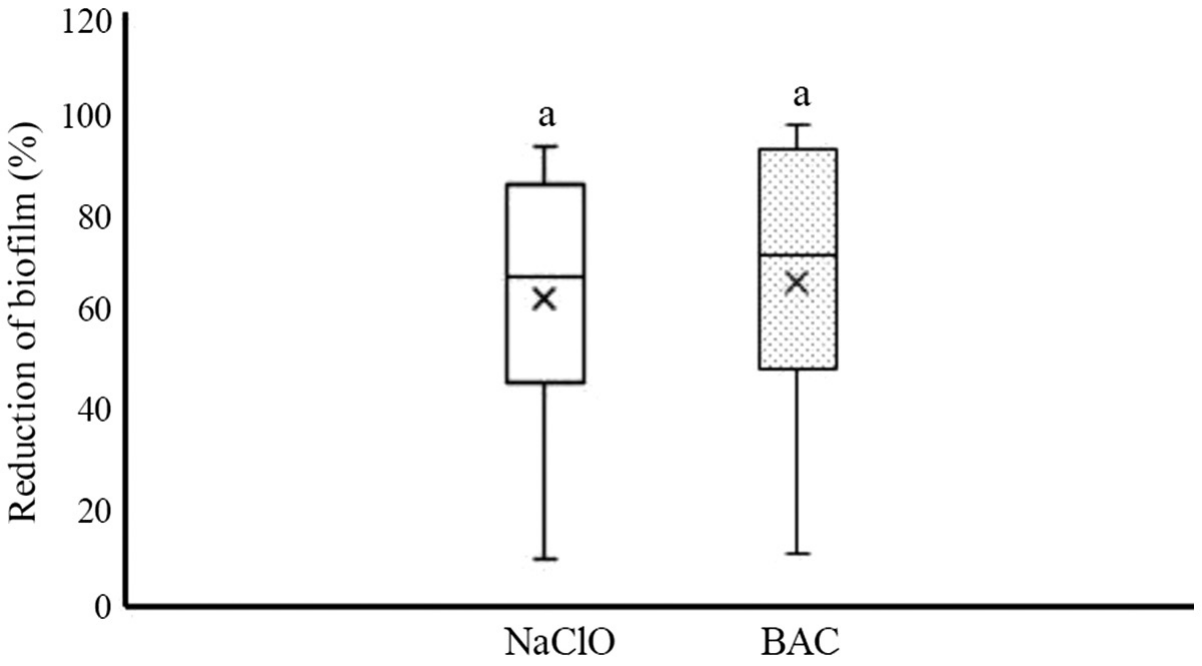
Reduction in *Salmonella* biofilm treated by sodium hypochlorite (NaClO) and benzalkonium chloride (BAC). Reductions that share a common letter are not significantly different (*p* > .05). Boxes show data within the first and third quartile (interquartile range, IQR), central horizontal lines show medians, x marks show means, and whiskers show the lowest data point within 1.5 IQR of the first quartile, and the highest data point within 1.5 IQR of the third quartile.

**FIGURE 7:**
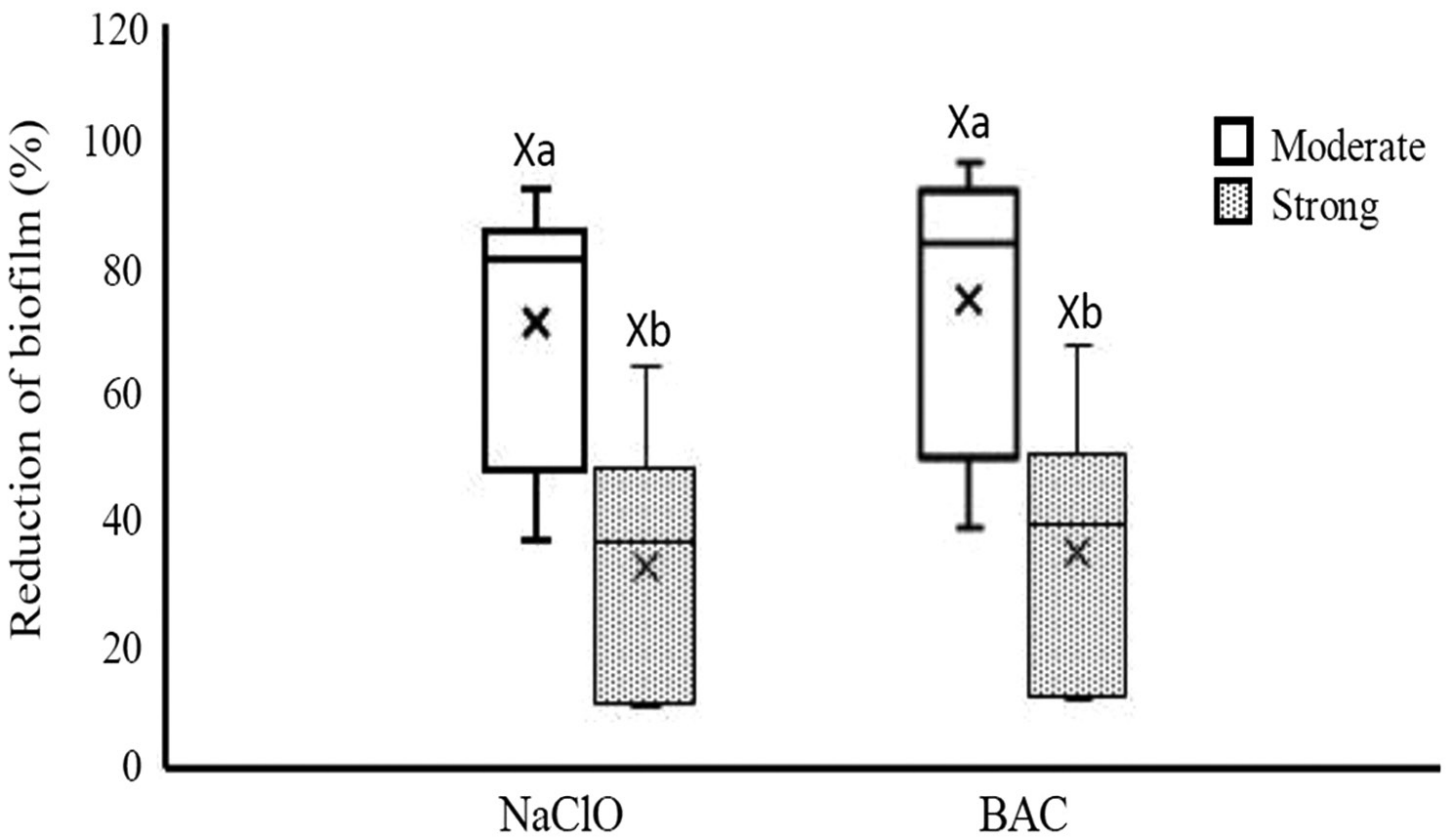
Reduction in biofilms of moderate and strong biofilm producers of *Salmonella* isolates treated by NaClO and BAC. Different small letters indicate significant differences (*p* < .05) in median reductions within a disinfectant. Different capital letters indicate significant differences (*p* < .05) in median reductions between disinfectants. Boxes show data within the first and third quartile (interquartile range, IQR), central horizontal lines show medians, x marks show means, and whiskers show the lowest data point within 1.5 IQR of the first quartile, and the highest data point within 1.5 IQR of the third quartile.

Evaluation of the effect of NaClO and BAC on biofilm removal was performed in a 96‐well microplate. The rate of reductions of *Salmonella* biofilm after treatment by NaClO and BAC ranged from 9.96% to 93.36% and 10.84% to 97.70%, respectively (Figure 6). The median percentage of reductions for NaClO and BAC were 66.8% and 70.98%, respectively, but there was no significant difference between the median reductions of two disinfectants (*p* < .05). Besides, the rate of reductions in biofilm treated with disinfectants was statistically analyzed according to the source of the isolation, serotypes, biofilm formation ability, and antibiotic resistance pattern. The results showed that the median reduction was not related to the source, serotypes, and antibiotic resistance pattern (*p* > .05). The reduction rates of moderate and strong biofilm producers were from 36.77% to 93.36% and 9.96% to 65.03% after treatment by NaClO, and from 38.62% to 97.70% and 10.84% to 68.19% after treatment by BAC, respectively (Figure 7). The median reductions for moderate and strong biofilm producers were 82.13% and 36.77% after treatment by NaClO, and 83.23% and 39.29 after treatment by BAC, respectively. Then, the median reduction of moderate biofilm producers was significantly (*p* < .05) higher than that of strong biofilm producers, in both types of disinfectants.

Moreover, an attempt was carried out to find any correlations among resistance to treatments (thermal, acidic, and alkaline) as well as between resistance to the treatments and resistance to the disinfectants. Pearson correlation coefficient (*r*) values between reductions after acidic and thermal, acidic, and alkaline, and thermal and alkaline treatments were 0.49, 0.07, and 0.23, respectively. The correlation between acidic and thermal treatment was significant (*p* < .05). Meanwhile, *r* values between reductions of the suspension of isolates after treatment by BAC and acidic, BAC and thermal, BAC and alkaline, NaClO and acidic, NaClO and thermal, and NaClO and alkaline were 0.13, 0.22, −0.12, 0.12, 0.03, and −0.7, respectively. Then, the correlation between NaClO and alkaline treatment was significant (*p* < .05). *R* values between resistance to treatments (thermal, acidic, and alkaline) and antibiotic resistance were −0.02, −0.11, and −0.12, respectively. Also, *r* values between resistance to treatments (thermal, acidic, and alkaline) and biofilm formation capacity were −0.23, −0.16, and −0.13, respectively. Then, the correlation between resistance to treatments (thermal, acidic, and alkaline) and antibiotic resistance or biofilm formation capacity was not significant.

## 4 DISCUSSION

In this study, the relative response of *Salmonella* serotypes (isolated from beef, mutton, and meat contact surfaces) to treatments applied in the meat industry, including thermal (60°C for 2 min), acidic (5% lactic acid, pH 2.9 for 30 min), and alkaline treatment (pH 11 for 60 min) was evaluated. The sensitivity of the isolates and their biofilms to selective disinfectants was also tested.

The results showed that *Salmonella* isolates had the highest susceptibility to acidic treatment, followed by thermal and alkaline treatments (Table 2). However, there was no significant difference in resistance among *Salmonella* strains isolated from different sources (beef, mutton, and meat contact surfaces) to thermal, acidic, or alkaline treatment (Figure 1). In other words, the response of *Salmonella* isolates to the treatments did not relate to their isolation source. In general, the isolates were significantly more sensitive to thermal and acid treatments than alkaline treatment. Besides, no significant difference was found among *Salmonella* serotypes susceptibility to none of the treatments. In a similar work, it has been shown that the isolates were more sensitive to acid and heat treatments than alkaline treatment and their responses were independent from the isolation source and serotype (Gill et al., 2019). In consistent with our findings, another study indicated that the response of strains to acidic and heat treatments had not relevance to their sources, serotype, or antibiotic resistance profile (Lianou & Koutsoumanis, 2013). Similarly, it has been reported that the response of *Salmonella* strains to the acidic and heat treatments was not correlated with strain source and serotype, while multi‐antibiotics resistant *Salmonella* strains showed higher resistance to HCl (Wang et al., 2021). Also, in examining the response of *E*. *coli* to different treatments, the response of *E. coli* to heat and acidic treatments was not related to its isolation source and serogroup (Gill et al., 2019; Lee et al., 2012; Liu et al., 2015). In several studies, unlike our results, low variability has been reported for *Salmonella* heat resistance compared with acid resistance (Gill et al., 2019; Lee et al., 2012). However, in consistent with our findings, another study showed a large variability in heat resistance among *Salmonella* populations; but, unlike our study, no correlation between resistance to heat and acid treatments was mentioned (Guillén et al., 2020). To the best of our knowledge, data on the response of *Salmonella* to alkaline stress are very scarce in literature. In accordance with our results, only one study has reported the highest variability in *Salmonella* resistance to alkaline treatment compared with acidic and heat treatments (Gill et al., 2019). In agreement with the results of the current study, a previous study reported that heat treatment reduced the number of *S*. Typhimurium in beef from 1 to 2 log CFU/ml at 60°C (Sarjit et al., 2019). It was recently reported that heat treatment (70°C for 5 min) could significantly reduce *Salmonella* count in a meat juice model (Sarjit et al., 2021). Furthermore, several studies have shown that *Salmonella* cell membranes are highly permeable to lactic acid (Alakomi et al., 2000; Wang et al., 2014; Wang, Chang, et al., 2015); therefore, it is one of the organic acids that decontaminate carcasses by disrupting cellular regulation (Yeh et al., 2018). Several other works have demonstrated the effectiveness of lactic acid on *Salmonella* reduction at different concentrations (Carlson et al., 2008; Chaine et al., 2013; Özdemir et al., 2006). However, some other studies reported that the acidic treatment could not significantly decrease *Salmonella* count (Yeh et al., 2018). Such differences in reported results may be due to type of strains studied and the type of acid used (Guillén et al., 2020).

It was shown that BAC and NaClO are effective disinfectants in destroying biofilms of foodborne pathogens (Yuan et al., 2021). Similar to the previous study, disinfectant susceptibility was observed with less deviation for the suspension of isolates compared with biofilm reduction, and the range of reduction obtained from microplate assay was higher than that of agar well diffusion (Lim et al., 2017). Besides, Lim et al. (2017) had shown that NaClO was relatively more effective than BAC to inhibit bacterial growth and biofilm removal (Loretz et al., 2011). In contrast, in the current study, no significant difference was observed between the median reductions caused by two disinfectants. Another study noted that NaClO is ineffective in removing bacterial biofilms, which contradicts our results. However, the results of BAC effects were similar in both studies (Ueda & Kuwabara, 2007). This difference in the results of the studies may be due to differences in temperature of biofilm formation or differences in the biofilm formation ability of each strain.

## 5 CONCLUSIONS

The findings of present study showed that acidic and thermal treatments are generally more effective decontamination interventions than alkaline treatment to control *Salmonella*. In addition, a relationship was found between susceptibility to alkaline treatment and antibiotic resistance pattern and that MDR isolates were more resistant to alkaline treatment than not‐MDR isolates. Also, *Salmonella* isolates with strong biofilm formation capacity were more resistant to disinfectant than other isolates. Taken together, these results suggest that effective treatments such as acidic treatment and appropriate concentrations of disinfectants could be used to control *Salmonella*.

## CONFLICT OF INTEREST

The authors declare that there is no conflict of interest.

## Data Availability

The data that support the findings of this study are available from the corresponding author upon reasonable request.
